# *Nautilus pompilius* Life History and Demographics at the Osprey Reef Seamount, Coral Sea, Australia

**DOI:** 10.1371/journal.pone.0016312

**Published:** 2011-02-10

**Authors:** Andrew J. Dunstan, Peter D. Ward, N. Justin Marshall

**Affiliations:** 1 School of Biomedical Science, University of Queensland, Brisbane, Queensland, Australia; 2 Department of Biology, University of Washington, Seattle, Washington, United States of America; 3 Queensland Brain Institute, University of Queensland, Brisbane, Queensland, Australia; University of Aberdeen, United Kingdom

## Abstract

Nautiloids are the subject of speculation as to their threatened status arising from the impacts of targeted fishing for the ornamental shell market. Life history knowledge is essential to understand the susceptibility of this group to overfishing and to the instigation of management frameworks. This study provides a comprehensive insight into the life of *Nautilus* in the wild. At Osprey Reef from 1998–2008, trapping for *Nautilus* was conducted on 354 occasions, with 2460 individuals of one species, *Nautilus pompilius*, captured and 247 individuals recaptured. Baited remote underwater video systems (BRUVS) were deployed on 15 occasions and six remotely operated vehicle (ROV) dives from 100–800 m were conducted to record *Nautilus* presence and behavior. Maturity, sex and size data were recorded, while measurements of recaptured individuals allowed estimation of growth rates to maturity, and longevity beyond maturity. We found sexual dimorphism in size at maturity (males: 131.9±SD = 2.6 mm; females: 118.9±7.5 mm shell diameter) in a population dominated by mature individuals (58%). Mean growth rates of 15 immature recaptured animals were 0.061±0.023 mm day^−1^ resulting in an estimate of around 15.5 years to maturation. Recaptures of mature animals after five years provide evidence of a lifespan exceeding 20 years. Juvenile *Nautilus pompilius* feeding behavior was recorded for the first time within the same depth range (200–610 m) as adults. Our results provide strong evidence of a *K*-selected life history for *Nautilus* from a detailed study of a ‘closed’ wild population. In conjunction with population size and density estimates established for the Osprey Reef *Nautilus*, this work allows calculations for sustainable catch and provides mechanisms to extrapolate these findings to other extant nautiloid populations (*Nautilus* and *Allonautilus* spp.) throughout the Indo-Pacific.

## Introduction

Growth rate, maturation time, longevity and population demographics are crucial information used within management frameworks such as the IUCN Red List and CITES (The Convention for International Trade in Endangered Species) to determine the status of species stocks [1], [2]. Life history knowledge of nautiloids is essential to the conservation management of this group as demand from the ornamental shell trade worldwide and restrictive habitat preferences contribute to what, at least in some geographic regions, appears to be their rapid decline [3]. From a world-wide oceanic distribution of nautiloids and their extinct predecessors over hundreds of millions of years, now only one to four species of *Nautilus* (taxonomy remains uncertain) and one separate genus, *Allonautilus*, represent the taxon in a distribution restricted to deep-water tropical habitats of the Indo-Pacific [4]. To date, there are five recognised nautiloid species *Nautilus pompilius* Linnaeus, *N. belauensis* Saunders, *N. macromphalus* Sowerby, *N.stenomphalus* Sowerby and *Allonautilus scrobiculatus* Lightfoot [*4*]. Current knowledge of *Nautilus* ecology is gleaned through an amalgamation of studies of life history and demographic parameters, using a range of methods and investigating different species, geographic morphs and geographically isolated populations of *Nautilus*.

Nautiloid populations in Australia, Palau, Papua New Guinea, Indonesia, Philippines, Fiji, Vanuatu, American Samoa and New Caledonia have been captured and studied. Field study and capture methods have generally relied on trapping of individuals from their deep water habitat but also include observations by in-situ deep camera deployment. All of these studies demonstrated a preponderance of males and mature animals with fewer sub-mature and immature specimens and a low percentage of juveniles. Mature size varies widely from 115 to 240 mm [5] between geographically distinct populations of *Nautilus* while sexual dimorphism distinguished by larger males is consistently reported [5], [6].

A *K*-selected life history of low fecundity, slow growth, late maturity and long life span for *Nautilus* has been supported by a range of studies from analysis of dead shells to long-term aquarium observations and trapping, mark and recapture studies in nature. Growth rates and lifespan have been estimated through growth lines on shells, chamber partial gas pressures [7], shell weight increases [8], radiographic measurement [9], [10], direct growth in captivity [9], [10], [11], [12], [13], measurement of recaptured animals in the wild [14] and radiometric analysis of shells [12], [15], [16]. Most growth rate estimates have used one or both of two different measures; those of circumferential growth and of chamber formation times.

Only one study has recorded growth rates in the wild by using recaptured individuals [14] and while individual numbers were low (4 immature and 7 animals in total) the results provided values consistent with radiometric and geochemical studies of the shell [15]. Aquarium studies uniformly showed higher growth rates than those observed in nature and these overestimation of growth rates were thought to be due to due to different conditions of food availability, temperature and energy expenditure to those in nature or the different constant ambient pressure of growth in surface aquaria compared to the daily variation of much greater pressures experienced in natural deep water habitats.

To date there is no direct observation of egg laying, depth of egg deposition or embryonic development of eggs in free-ranging populations. It has only been possible to study these earliest stages of *Nautilus* life in surface aquaria and reports of *Nautilus* egg laying in captivity have recorded low numbers of eggs being laid throughout the year [17], [18], [19]. Observations of embryonic development indicate a period to hatching stage of slightly less than 12 months, resulting in a juvenile with seven chambers and of approximately 26 mm shell diameter [17], [18], [19], [20]. With no observations of egg laying locations or early juvenile habitat these aspects have relied on analysis of shell septa by radiometric and geochemical methods to determine the depth at which embryonic development occurs. Differences in the radio-isotopic composition of the first seven septa in comparison to post hatching septa infers an embryonic period in relatively shallow water of around 100 m in contrast to post hatching distribution and movements between 100 and 700 m [21], [22], [23], [24]}. Capture of juveniles by trapping have been rare and early juveniles only reported twice [25], [26].

Slow growth, slow reproduction, lack of dispersal options and low population numbers suggest sensitivity to over-exploitation. However, in spite of these “red flags”, at present, *Nautilus pompilius* and other *Nautilus* and *Allonautilus* species are not included within the IUCN Red List (IUCN 2009), sustainable fishing rates have not been addressed in the *Nautilus* fisheries of the Indo-Pacific and broad-scale nautiloid conservation plans are not in place.

We aim to provide a comprehensive description of *Nautilus* growth, age and sex distribution and life history in a single wild population for the first time. The results now enable a much more informed approach to decision making about *Nautilus* and *Allonautilus* fishery management and conservation protocols for the long term future. The mysteries of egg laying locations, the low percentage of juveniles in a population and habitat and behavior of post hatching juveniles has not been solved: however the methodological background and access to deep camera, ROV, deep re-breather diving to 150 m and manned submersible equipment and techniques at Osprey Reef in the immediate future should allow a completely new approach to solving this conundrum.

## Results

### Sampling summary

**Figure 1:**
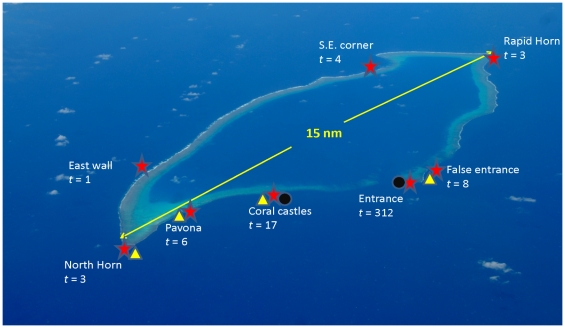
Nautilus sampling at Osprey Reef by trapping and Baited Remote Underwater Video Systems (BRUVS) and Remotely Operated Vehicle (ROV) camera records. Nautilus trapping was conducted at eight sites (red stars, *t* = number of samples) around Osprey Reef totalling 354 samples of which 92% were at the Entrance site. (BRUVS) deployments (black circles) were undertaken at two sites while (ROV) dives (yellow triangles) were conducted at four sites.

Data was collected between 8/06/1998 and 9/12/2008 during which 354 traps were set and recovered at eight sites around the perimeter of Osprey Reef (Fig. 1). A detailed Osprey Reef location and bathymetric map is available from previous work [27].

Of the 2460 individuals captured, 247 were recaptured once, 17 recaptured twice and three recaptured three times. A single species, *Nautilus pompilius*, was captured. Maturity records for 1912 individuals were taken, the sex of all individuals was recorded where possible and in all cases for the 956 mature individuals. Shell length and inter-ocular apertural width were measured for 2067 individuals and digital images of the left, right and dorsal sides taken. Growth since previous capture was measured for all recaptures and actual growth recorded from 37 individuals which were immature at the time of first capture.

**Figure 2:**
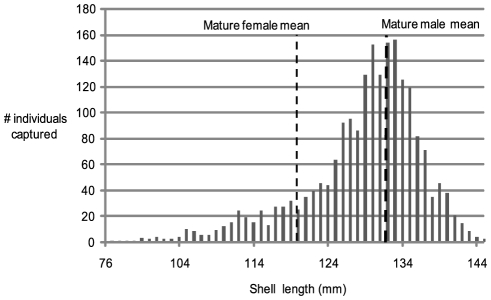
Size frequency distribution for *Nautilus pompilius* at Osprey Reef. Size frequency records for 2067 individuals range from 76 to 145 mm with a mean of 128.6±28.01 mm. Mature male and female mean shell lengths are shown.

**Figure 3:**
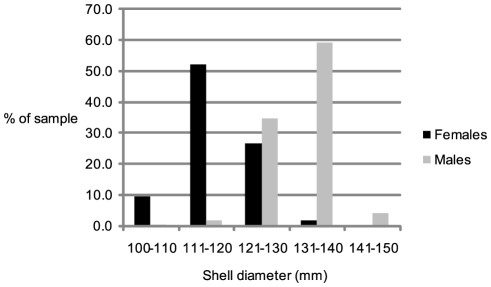
Sexual dimorphism in mature male and female *Nautilus pompilius*. Males were larger (light bars: mean shell diameter = 131.9±2.6 mm, apertural shell width = 67.9±5.2 mm, N = 870) than females (dark bars: mean shell diameter = 118.9±7.5 mm, apertural shell width = 61.5±4.7 mm, *n* = 86). Maturation occurred at sizes varying±11% of the mean maximum shell diameter.

2067 captured individuals were measured for maximum shell diameter. The results show a distribution dominated by mature or near-mature animals (Fig. 2). This is a left-skewed distribution due to size based sexual dimorphism, minimized by the low percentage of females in the sample population (Fig. 3). Note that juveniles of less than 80 mm shell diameter (<50 mm shell width) were able to escape the trap mesh and are under-estimated by this sampling method.

### Sexual dimorphism

Analysis of mature *Nautilus* data showed that males were larger (131.9 mm) and more abundant (89.5%) than females (118.9 mm) (Fig. 3). Ratio of shell length to inter-ocular apertural shell width was 1.94 for both sexes. Three mature males and three female shells measured had 27 chambers (20 post neopolic margin) and one male possessed 28 chambers. The ventral circumference to shell length ratio (mean = 3.403±0.029) was consistent for all specimens and used to calculate circumference of recaptured individuals for age estimation.

### Seasonal and depth effects on capture rates

**Figure 4:**
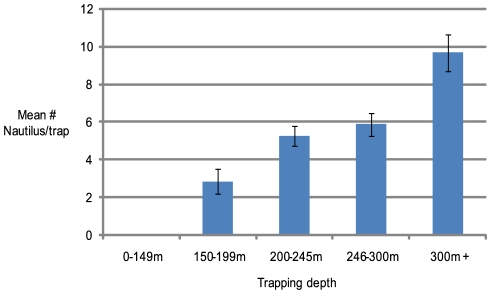
Comparison of *Nautilus* capture rates with trapping depth. The influence of depth on capture rates was analyzed for 271 trapping events spread throughout all months of the year. The most effective capture depths were around 300 to 350 m, while no captures were recorded from 18 trapping efforts at depths <150 m. Error bars (±1 standard error of the mean) are shown.

Comparison of mature male to female ratio between 3 month seasons showed minimal effect; varying from 86.3% during December–February to 93.1% during June–August (Table S1). Overall capture rate seasonality is relatively constant from 5.7 (Dec–Feb) to 7.9 trap^−1^ (Jun–Aug) (Fig. S1). Analysis of trapping depth effects on capture rate show trapping at 300 to 350 m to be the most productive with a mean capture rate of 10.1 *Nautilus* per trap (Fig. 4). No *Nautiluses* were captured shallower than 150 m although one was sighted during a BRUVS deployment to 100 m.

### Size and maturity distribution within the Osprey Reef Nautilus population

The ratio of mature animals in both large and small mesh traps exceeds that of immature and juvenile individuals. At around 90 mm shell length the color lines at the shell aperture were replaced by a fully white ventral apertural margin, used in previous studies to indicate post juvenile stages.

Trapping methods resulted in the capture of three juvenile *Nautilus* with shell lengths of 76, 87.5, and 89.5 mm, captured from 375, 265 and 330 m depths respectively. Seven other individuals with shell lengths <100 mm were captured from depths between 200 and 330 m. Small mesh traps set between 90–460 m in all seasons on 41 occasions failed to capture any juvenile *Nautilus* (Table 1 and Table S2).

**Table 1:** Mature, immature and juvenile capture ratios.

Trap type	Sample #	Juvenile	Immature	Mature	Total
Large mesh	354	3	807	1102	1912
		0.002%	42%	58%	100%
Large mesh ‘twin’ trap	41	0	102	169	271
		0%	38%	62%	100%
Small mesh ‘twin’ trap	41	0	0	0	0

Trapping data for all 354 trap samples shows a majority of mature individuals (58%). The large traps used in conjunction with ‘piggy-backed’ small mesh traps show a similar ratio of mature to immature individuals, while the small mesh trap caught only prawns and amphipods.

Baited remote underwater video system (BRUVS) records from 15 deployments and 65 hours of observations [28] show a total of 86 individual *Nautiluses* were observed during this time, including only one juvenile estimated at around 90mm shell length.

**Figure 5:**
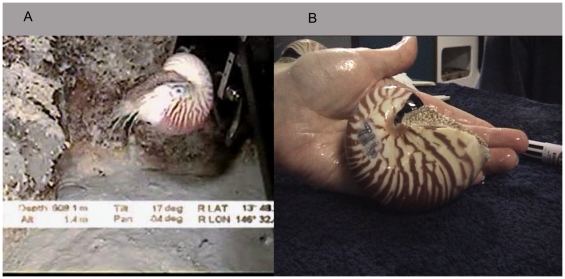
Juvenile *Nautilus pompilius*. A: Juvenile *N. pompilius* about 70 mm shell length filmedfrom the ROV actively foraging at 608.1 m at North Horn, Osprey Reef. B: *Nautilus* # 716 with shell length of 89.5 mm captured by trap from 330 m. Note the cessation of color banding lines at the apertural margin and the start of this area becoming a fully white region, indicative of final juvenile stage.

Remotely operated vehicle (ROV) observations during six dives at four sites on Osprey Reef (Fig. 1) provided a total of 29.05 hours of video footage and still images from 100 to 800 m depth during daytime only. Sightings of *Nautilus* were only recorded deeper than 420 m with most between 450–650 m. All *Nautiluses* were actively moving in foraging mode and many demonstrated some attraction to the ROV. Of the 48 individuals recorded five were juveniles, sighted between 490 and 608.1 m (Table 2 & Fig 5).

**Table 2:** ROV *Nautilus* sightings at Osprey Reef.

Depth (m)	0–300	300–400	400–500	500–600	600–700	700+	Total
# Nautilus	0	0	7	23	17	1	48
# juveniles	0	0	1	3	1	0	5
Search time (hrs)	4.25	3.83	7.92	7.4	4.57	1.08	29.05

ROV sightings of Nautilus during daylight hours in relation to search time at depth are shown. Sightings were only recorded deeper than 489 m, with most between 500–650 m, including juveniles sighted between 490 and 608 m.

### Growth rates

**Figure 6:**
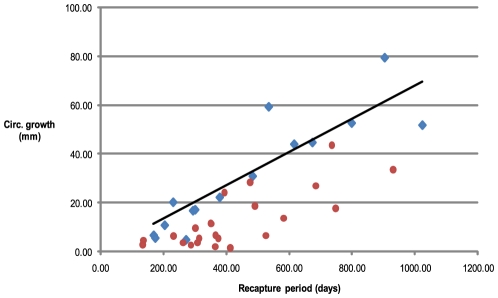
Circumferential growth rates of *Nautilus pompilius* at Osprey Reef. *Nautilus* which were immature at both first capture and recapture (diamond symbol, n = 15) were used to calculate growth rate. A linear regression showed a growth rate of 0.068 mm/day while mean growth rate = 0.0614±0.023 mm day^−1^). Individuals which were immature at first capture but matured during the time before recapture (circle symbol, n = 23) showed varied growth rates, all <0.0614 mm day^−1^.

Only 15 of the 247 recaptured *Nautiluses* were immature at initial capture and recapture times and therefore able to provide accurate growth estimates with a mean of 0.0614±0.023 mm day^−1^) and a range between 0.018 and 0.111 mm day^−1^. There was no significant difference between growth rates for males and females (Table 3 & Fig. S2). A further 23 individuals which were immature at first capture but matured during the recapture period also had growth rates calculated, knowing they should be less than maximal given an unknown time for maturation and growth cessation. Three of these animals had growth rates approximating the immature mean of 0.0614 mm day^−1^ while the remaining 20 animals' growth rates were lower, ranging from 0.009 to 0.039 mm day^−1^ (Fig. 6).

**Table 3:** Recaptured *Nautilus* measurements, growth rate and age estimates.

Animal	M/F	Recapture interval (days)	Shell length (mm)	Initial circ. (mm)	Circ. Growth (mm)	Growth rate (mm/day)	Relative annual growth (%)	Age estimate (yrs)
1616	m	169	119	404.6	6.76	0.040	3.6	23.6
1179	m	174	122	414.8	5.63	0.032	2.6	30.0
1363	m	272	123	418.2	4.92	0.018	1.6	53.1
1347	m	294	127	431.8	16.82	0.057	4.9	18.6
477	m	484	118	401.2	30.93	0.064	5.8	16.0
397	m	535	107	362.1	59.30	0.111	11.5	9.3
697	m	616	125	423.3	44.00	0.071	6.2	15.8
274	m	904	106	361.4	79.38	0.088	8.8	12.1
1912	m	799	112	379.4	52.64	0.066	6.5	15.5
723	m	1024	112	380.8	51.80	0.051	4.8	19.8
1992	f	301	114	387.6	17.21	0.057	5.3	16,5
2156	f	204	131	445.4	10.88	0.053	4.4	20.4
1426	f	379	118	401.2	22.27	0.059	5.3	16.8
1535	f	231	124	421.6	20.27	0.088	7.6	12.2
1928	f	674	102	346.8	44.70	0.066	6.9	13.8

Size and growth data for ten male and five female recaptured *Nautiluses* which were immature at both first capture and recapture times. The mean circumferential (circ.) growth rate of 0.0614±0.023 mm day^−1^; range = 0.018 to 0.111 mm day^−1^) provides an age estimate of 20+ years.

### 3.6 Age estimation

Age estimates for *Nautilus pompilius* of Osprey Reef to reach maturity take into account pre-hatching periods of around 315 days to reach approximately 26 mm diameter and 80 mm ventral circumference. Growth from hatching to maturity is estimated from mean growth rates of immature recaptured *Nautilus*. Using the mean growth rate and assuming linear growth of 0.0614mm day^−1^, the estimated time to reach maturity for males and females of mean mature shell length is 16.5 years and 14.5 years respectively. Age to maturity is also calculated for each individual in Table 4. Using the maximum recorded growth rate of 0.111mm day^−1^ provides much reduced mature age estimates of 9.1 years for males and 8.01 years for females.

**Table 4:** *Nautilus* size, septa number and growth rate comparisons.

Location	Species	Study	Mean mature shell length (mm)	# septa	Circ. Growth rate (mm/day)	Relative annual growth rate (%)	Final stage
Osprey Reef, Australia (this study)	N.pomp	F	130.7	27	0.061	6.3	I - S
Manus, PNG [12]	N.pomp	R	176.6	-	0.12	8.3	I - M
Philippines [10], [12], [13]	N.pomp	A	174	35	0.124, 0.23, 0.24	8.7, 16.1, 16.8	I - M
Philippines [16]	N.pomp	R	174	35	0.21	14.7	I
Palau [15]	N.belau	R	200–204	35–37	0.12	7.1	I - M
Palau [14]	N.belau	F	200–204	35–37	0.12	7.1	I - M
Palau [10]	N.belau	A	200–204	35–37	0.24	14.2	I - M
New Caledonia [16]	N.macro	R	165	31	-		I - M
New Caledonia [11]	N.macro	A	165	30–32	0.14	10.5	I
New Caledonia [8]	N.macro	A	165	31	0.25	18.75	I
N.Sulu Sea, Indonesia [5]	N.pomp	F	115.6	26–28	-	-	-
GBR, Australia [38]	N.pomp	F	159.8	-	-	-	-
New Ireland, PNG [38]	N.pomp	F	173.3	-	-	-	-
Port Moresby, PNG [5]	N.pomp	F	151.8	27–30	-	-	-
Lae, PNG [5]	N.pomp	F	146.1	-	-	-	-
Fiji [35]	N.pomp	F	133–141.6	28–29	-	-	-

Past *Nautilus* studies where mature shell length was measured and includes data for septa number, growth rate and stage of maturity where determined are summarized. The type of study is indicated by: F = field, R = radiometric/geochemical, A = aquarium.

**Figure 7:**
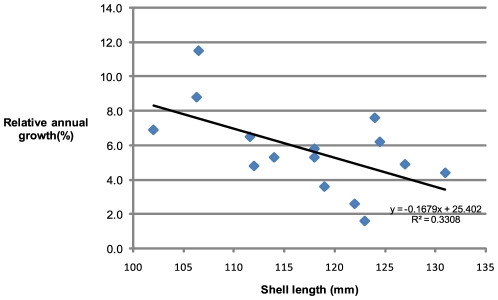
Relative annual growth rates compared to shell length at first capture. The relative annual growth rate of each individual immature *Nautilus* when compared with shell length at time of first capture is indicative (R^2^ = 0.3308) of growth rates decreasing as maturity is approached.

Relative annual growth provides a measure of growth rate as a function of body size; an especially useful tool for comparing *Nautilus pompilius* from geographically isolated regions where mature shell sizes differ markedly.
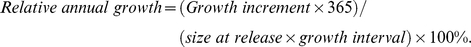
It can also be used to compare growth rates of different age/size groups within a population. In this instance there is an indication (R^2^ = 0.3308) of higher relative growth rates in younger Nautilus with this rate decreasing as animals approach mature size (Fig 7).

### Longevity

**Figure 8:**
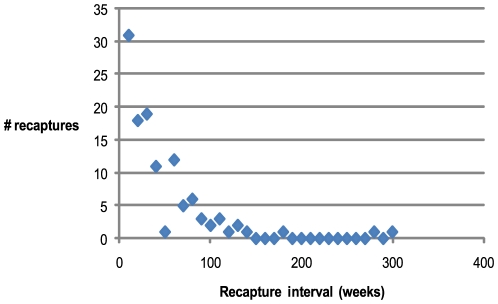
Mature *Nautilus pompilius* longevity after first capture. Maximum time between capture and recapture of an already mature animal with no recorded growth was 5.3 years with a mean recapture period of 48 weeks.

While aquarium hatching times and initial chamber formation can estimate pre-hatching growth periods and wild growth rate information provides age estimation to maturity there is still the question of longevity beyond maturity. The mark and recapture records of individuals in this study show a maximum recapture period of 5.3 years (two specimens) and numerous specimens from 2–3.5 years after previously being captured as mature animals (Fig. 8).

## Discussion

### The demographics of the *Nautilus pompilius* population at Osprey Reef

Demographic and life history studies of *Nautilus* are well documented in the literature but to date have a fragmented pattern of investigation. This has required the different parameters of mature size, age class structure, embryonic period, growth rate, maturation time and lifespan to be combined from various field, captive and radiometric studies to provide a complete picture. It is generally accepted that captive studies over-estimate growth rates and while providing valuable insight to *Nautilus* life history, and providing the only data relating to embryonic growth and hatching, are only indicators into what occurs in nature. This study provides the most comprehensive insight into the growth, life history and demographics of a wild population of *Nautilus pompilius* to date.

*Nautiluses* have no larval dispersal stage and are benthic organisms restricted to depths shallower than their reported shell implosion depth of 800 m. Osprey Reef is an oceanic seamount surrounded by 2000 m depths and harbors a geographically isolated population of *N.pompilius*. Past studies have shown the genetic variation in broadly separated geographic populations and morphological descriptions detail the variability in mature shell length, color patterns, radula structure and hood and tentacle sheath form of *N. pompilius* [*29*]. More recent genetic characterization points to a more realistic taxonomic designation of just three *Nautilus* species, *Nautilus pompilius*, *N.macromphalus*, and *Allonautilusscrobiculatus*, with *N. belauensis*, *N. stenomphalus* and *N. repertus*, along with other geographically segregated clades of *N. pompilius*, as separate subspecies [*4]*, [30**].

In characterizing the demographics of *Nautilus pompilius* of Osprey Reef we find similar aspects of sexual dimorphism, distribution of sexes and age classes and vertical depth regimes to populations investigated in other locations. Osprey Reef *Nautilus pompilius* are the second smallest yet described at a mean shell length of 130.7 mm, flanked on either end by the diminutive Sulu Sea clade of 115.6 mm and northwest Australian specimens, earlier reported as *N. repertus*, of 240 mm [5] shell length. Sexual dimorphism with larger males and a mature male∶female size ratio of 1.11 is similar to other populations with ratios of 1.02 to 1.14. The dominance of males in the population is also consistent throughout past reports, with Osprey Reef data showing 89.5% males, in broad agreement with data from all other locations where ratios vary from 60 to 94% males. The sole instance of female dominance is one Philippine study with a ratio of 32% males from a small sample size of 32 individuals [31]. Table S3 summarizes these comparative findings and references the studies which produced them.

Haven [32] reported a seasonality in female presence in traps but this study found no such indications (Table S1) with male capture rates consistent between 86.3 to 93.1% in pooled three monthly seasons.

Catch rate variation with sampling depth provided similar results to other studies [26], [29] with the exception of those in Palau [33] and Tanon Straits, Philippines [32]. This variation may be related to the optimal feeding zone of a particular habitat related to the bathymetric topography and substrate type. At Osprey Reef the vertical rocky walls descend to around 250–300 m below which there is a sloping silty substrate perhaps more suited to scavenging feeding behavior. Vertical migration studies at different locales provide more evidence to support this hypothesis [27].

### Size frequency distribution

The understanding of age demographics of *Nautilus* populations is limited by the lack of observations and knowledge of *Nautilus* juveniles. Capture records are minimal and in-situ observations rare. *N. pompilius* at Osprey Reef are predominantly mature (58%) which equates with other localities, all reporting >50% mature individuals with the exception of one of three Fijian studies reporting 16% [34] and Saunders' American Samoan sample documenting 28.2% [35] mature animals. Immature and sub-mature animals dominate the remaining 42% of individuals sampled at Osprey Reef, as seen in other studies. The low number of juveniles in this study is consistent with previous trapping studies where the highest juvenile catch rate was 5% [14]. This may be a sampling bias related to mesh and entrance size allowing preferential escape by juveniles.

Remote cameras and ROV observations can provide a more accurate, albeit lower sampling intensity, than the trapping studies. The juvenile records from traps, BRUVS and the ROV show active feeding at depths ranging from 265–610 m demonstrating that juveniles do indeed actively inhabit the same range as adult animals. The sighting of 5 juveniles from a total of 48 individuals during ROV observations is an indication of higher levels of juveniles than represented in trapping studies and correlates with Saunders' deep camera photo-sequence data of five juveniles within 41 individuals observed [33]. Both these studies observed juveniles in their later stages of growth, approaching immature stages, and rarely have small post-hatching stage *Nautilus* been sampled in the wild. Future sampling at Osprey Reef will aim to diversify in sampling methods to attempt to answer the ongoing mystery of early juvenile location and behavior.

### Growth rates

*Nautilus* growth rate calculations have relied on three main methodologies; measurement of growth in recaptured animals in the field, aquarium studies measuring growth during captive periods, and radiometric methods analyzing shell septa. Growth studies and the data they have produced are summarized in Table 4 and Table S4.

This Osprey Reef study provides the largest dataset of growth rates for animals which were immature at both initial capture and recapture; 15 individuals, as compared to the only other wild study, by Saunders in Palau [14], where four immature recaptured *N. belauensis* provided growth measurements. Captive growth studies have been conducted on a range of *Nautilus* species or sub-species from a variety of locations and all authors temper their results with the caveats common to captive studies, where growth may differ to wild growth patterns due to the availability of a constant food source, reduced energy expenditure and zero predator and injury impacts. Captive growth rates would therefore be expected to exceed growth rates in nature and from all results available this is the case. Radiometric data provide a parallel means to determine wild growth rate. The analysis of shells from Saunders' Palau study by Cochran and Landman [15] correlate closely with wild growth rates measured from mark/ recapture studies with both resulting in calculated rates of 0.12 mm day^−1^ circumferential growth. Landman's results of 0.12 mm day^−1^ for *N.pompilius* from PNG also agree [12].

This study describes slower circumferential growth rates (0.061 mm day^−1^) of *Nautilus* individuals which are much smaller (130.7 mm) than those of Saunders' Palau subjects (0.12 mm day^−1^ and 200 mm mean shell length). The individuals which matured during the recapture interval all demonstrated growth rates less than that of the immature recaptures, as expected, and provide support to the overall growth rate calculations. Both Cochran's study (immature mean = 0.12 mm day^−1^, sub-mature mean = 0.085 mm day^−1^) and this work (Fig. 7) indicate growth rate slowing as animals approach maturity. The Osprey Reef data show no difference in growth rates between males and females (Fig. S2).

### Relative annual growth rate

To compare growth rates between *Nautilus* populations from different geographic areas and of differing maximum sizes a relative annual growth index was used. This reflects growth from hatching to maturity, requiring the subtraction of size at hatching, reported consistently at around 26 mm shell length and with seven completed chambers [36]. No circumferential size data are reported for hatchlings, and other growth studies, with the exception of Saunders', did not record mature shell circumference. The measures of total circumference and shell length from this study and that of Saunders do however allow a constant ratio to be calculated which can be used to convert circumferential growth rate to shell length growth rate and utilize the shell length data available for all growth studies (Table 4 and Table S4).
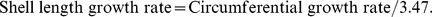
The conversion factor of 3.47 is averaged from measurements of this study and the study of Saunders, the only studies where total circumference and length have been measured from longitudinally dissected shells of individuals, with circ. / length means of 3.40±0.029 (n = 7, shell length range = 115–135 mm) and 3.54±0.047 (n = 14, shell length range = 177–212.8 mm) respectively. From this information, relative annual growth % can be calculated by the following equation:
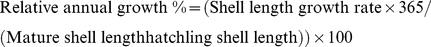



The results of relative growth rate estimates in the wild and radiometric studies show growth rates of the Palau [15], [37] (7.1%), PNG [12] (8.3%) and Osprey Reef (6.3%) *Nautiluses* to be very similar for sub-mature specimens, while the radiometric determinations of two juvenile specimens from the Philippines [16] resulted in relative growth rates of 14.7%. This higher growth rate for juveniles is in contrast to the captive studies by Westermann [10] showing growth rates from all age classes; juvenile, immature and sub-mature, to be around 8.7% (Table 4 and Table S4). These calculations indicate that larger maximum size may not necessarily mean longer lifespan for *Nautilus* from different geographic locales. They also point towards a constant growth rate after hatching until a decrease just prior to maturation and growth cessation, however more study must be conducted on juvenile growth rates to confirm constant growth.

Assuming circumferential growth rates of Osprey Reef *N. pompilius* to be constant from hatching to maturity, an estimated maturation time of 14.5 and 16.5 years for females and males respectively is achieved. This estimate is in close accord with the 14.5–17.2 years determined by Saunders [37], the only other wild study using mark and recapture techniques (Table S4) and which also assumed constant growth.

### Lifespan of *Nautilus pompilius*

Calculations of the lifespan of *Nautilus* from existing studies require the knowledge of timing for pre-hatching, post-hatching maturation and post maturity longevity times. This study provides estimates for *N. pompilius* of a post-hatching maturation time of approximately 15.5 years while previous aquarium studies show embryonic periods of between 269–362 days to reach 25–30 mm shell length and seven septa at hatching [17], [18], [19], [36].

Data from mark recapture at Osprey Reef provide the longest recorded time recorded for *Nautilus* longevity beyond maturity of 5.3 years for two specimens. Saunders recorded recapture of a mature individual four years after maturity [37]. Combining these data extends past captive estimates of 11–12 years [10] to indicate that *N. pompilius* has a lifespan in excess of 20 years.

Preliminary observations from our captures of *N. pompilius* from four different seamounts in the Coral Sea, separated by only a total of 200 nautical miles, indicate separate populations distinguished by mature size and color pattern variation. Given the similarity of the habitats this may prove to be a valuable testing area to investigate growth rate variability within a habitat type and compare this with a range of varied habitats such as those found in the deltaic areas of PNG, gently sloping regions of the Tanon Straits, Philippines and reef walls of Palau.

### Conclusions

This growth study and its associated population estimation [28] and movement pattern [27] of *N. pompilius* at Osprey Reef, Australia provide the most comprehensive understanding of *Nautilus* life history, demographics and behavior yet available for a single population. The data describe a population of low overall number and density, with a majority of males and mature individuals and a k-selected life history. All parameters point to an organism which is vulnerable to low levels of exploitation, as has been demonstrated through changes in catch per unit effort in Philippines *Nautilus* fishery studies [3]. This detailed knowledge of growth rates and population demographics should enable similar methodologies to be used to characterize *Nautilus* populations elsewhere, to define sustainable catch levels and to form the basis for sustainable management plans; conservation protocols and / or CITES listing for *Nautilus* in the future. They should also allow an informed basis for the IUCN Red List classification of *Nautilus* to further the conservation of this group.

## Materials and Methods

### Ethics statement

Research was conducted under permit from the Australian Fisheries Management Authority and with ethics approval from the University of Queensland Animal Ethics Committee.

### Capture methods

A barrel shaped trap (90×77 cm) constructed from wire mesh (75×50 mm mesh size), baited with chicken and set to an average depth of 300 m, was used to trap *Nautilus*. Sampling was conducted between 1998–2008 and throughout the year (with the exception of February only having one sample) with a total of 354 traps set and retrieved (Tables 1 & 2). On 41 separate occasions baited small mesh (30×30 mm) traps with small entrance funnels (90 mm diameter) were attached to the larger traps, specifically to sample for juveniles between 100 and 460 m depths. Sample sites were around the entire circumference of Osprey Reef but with 92% of sampling undertaken at the Entrance site (Fig. 1). Traps were deployed for 12 hours; set at dusk and retrieved vertically at dawn at 15 m min^−1^ with *Nautilus* immediately placed in a dedicated, dark refrigerated tank to contain the animals during the tagging process at temperatures between 16–19° C. Depth of trapping was determined by using measured rope length on all occasions and on 20 occasions attaching temperature-depth recorders to the trap. A linear relationship was found between rope length and depth and a constant factor then used to calculate depth of fishing for all trapping events.

### Tagging and recording of demographic parameters

**Figure 9:**
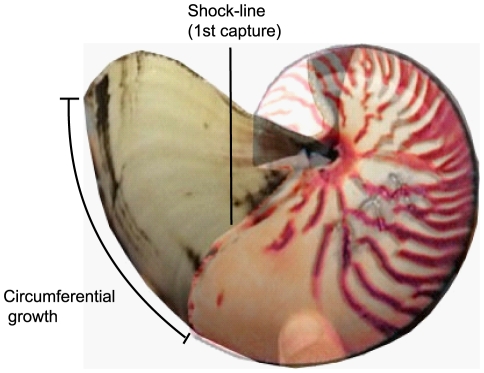
Growth in shell circumference and diameter of *Nautilus pompilius*. Initial capture image overlaid on recapture image of *Nautilus* #274 showing 2 ½ years of circumferential growth at a rate of 0.61 mm / day. The shock-line of initial capture can be seen on the recapture image and provides an easy method to check growth measurements.

All captured *Nautilus* were tagged by engraving the shell with an identification number which was then etched with permanent marker and covered with clear enamel. Each individual was photographed and maximum shell length (diameter) and aperture width at the ocular sinus were measured using vernier calipers accurate to 0.1 mm. Circumferential growth was measured for recaptured animals from the initial capture ‘shock line’ identified by Saunders [37]. Growth was later verified by comparing and overlaying initial and recapture digital images (Fig. 9).

Seven mature *Nautilus* (four males and three females) were sacrificed for laboratory studies and their shells measured for total ventral circumference, shell length and number of chambers. Constant values were observed for total ventral circumference to shell length ratio (3.403) and this constant used to calculate circumference for recaptured individuals.

Animals were recorded as mature (blackened and thickened apertural margin and accentuation of the ocular sinus), sub-mature (no black apertural margin which is sharp edged and with color stripes not extending to aperture) or juvenile (color stripes extending to aperture). Sex was determined by the presence of a spadix (male only), by the position of the jaw apparatus (central in females, offset to left or right in males), or by the presence of nidamental glands (female only), before release [32], [37]. These requirements resulted in sex determination for mature and near mature animals, but not for immature or juvenile individuals. Tags were always visible on recaptures but identity of each recapture was checked during later analysis using the unique ‘fingerprint’ of each individual shell pattern.

Maturity, sex and length records were limited to data where consistent methodology was used and identification of the parameter could be relied upon. Growth calculations were made from recaptured animals which were immature at time of first capture and for which photographic images were recorded.

To reduce the chance of predation by visual predators upon release, *Nautilus* were kept on board for 13 hours prior to release after dark. Animals were checked for neutral buoyancy and normal swimming and descent behavior during release by scuba at 30 m at the capture location, following the methods of Saunders [37].

### Baited remote underwater video system (BRUVS) observations

A T-shaped steel frame one meter in length had a *Nautilus* trap attached with opening facing away from the T-bar. A Sony CX12 camera in purpose built housing and an LED light of red, blue or white color were mounted on the T-bar. During December 2009 and May 2010, 15 BRUVS deployments were made; 14 at 300 m and one at 100 m with all except one daytime deployment being made from dusk onwards. Camera battery and memory chip capabilities limited recorded observation periods to between 4 and 7.5 hours per deployment. A total of 65 hours of BRUVS observations were analyzed for presence of juvenile *Nautilus*.

### Remotely operated vehicle (R`OV) observations

A Cherokee ROV capable to 800 m depths was deployed at Osprey Reef [28] for six daytime observations at four different sites for dives of between four to eight hours. Standard mini DV footage and still images using white halogen light sources were taken alongside real-time linked observer data recording. Records of depth and location of the ROV were then matched with image and written records for nautilus presence and maturity.

## Supporting Information

Figure S1 **Overall capture seasonality.** Capture seasonality was assessed for three month periods over five years. While a slight increase is apparent in June to August there is no evidence for any seasonal trend in capture rate. Error bars (±1 standard error of the mean) are shown.
(EPS)

Figure S2 **Growth rates of immature male and female** ***N. pompilius*** **.** A comparison of male (blue diamonds, y = 0.0687x) and female (red squares, y = 0.0648x) growth rates for Nautilus which were immature at both first capture and recapture times. No difference between male and female growth rates is observed.
(EPS)

Table S1 **Seasonality in ratio of mature male to female captures.** Capture numbers and percentage ratio of males to females during three month seasons of the year over five years are compared. Sample numbers were similar for each season and the % male value range from 86.3–93.1% did not vary greatly from the mean value of 89.5%. There is no evidence for seasonality in male or female capture bias.
(DOCX)

Table S2 **Small mesh trapping capture results.** Twin traps comprising one large mesh (75×50 mm) and one small mesh (30×30 mm) trap ‘piggy backed’ together were deployed on 41 occasions between 100 to 460 m and failed on all occasions to capture any juvenile *Nautilus*. The large mesh trap caught immature to mature *Nautilus* at a rate consistent with overall capture rates.
(DOCX)

Table S3 **Comparison table of** ***Nautilus*** **demographic studies.** A comprehensive summary of field study data for the *Nautilus* demographic parameters of shell length, number of septa, male: female ratio and percentage of mature individuals is provided. The data comes from a wide range of studies and locations and covers all extant species of *Nautilus* known to date.
(DOCX)

Table S4 **Comparison table of *Nautilus* growth rate studies.** Growth, embryonic development period and age estimation data are summarized from field (F), aquarium (A) and radiometric / geochemical (R) studies in the literature. Note the similarity of age estimates for both this study and that of Saunders, the only field-based studies of growth rate.
(DOCX)
