# Supplementary material for: Nautilus pompilius Life History and Demographics at the Osprey Reef Seamount, Coral Sea, Australia
Source: PLoS One. 2011 Feb 10;6(2):e16312. doi: 10.1371/journal.pone.0016312 (PMC3037366; doi:10.1371/journal.pone.0016312)
Supplement: Table S1 — Seasonality in ratio of mature male to female captures. Capture numbers and percentage ratio of males to females during three month seasons of the year over five years are compared. Sample numbers were similar for each season and the % male value range from 86.3–93.1% did not vary greatly from the mean value of 89.5%. There is no evidence for seasonality in male or female capture bias. (DOCX) [file pone.0016312.s003.docx]

**Table S1. Seasonality in ratio of mature male to female captures.**

|  | **Dec-Feb** | **Mar-May** | **Jun-Aug** | **Sep-Nov** | **TOTAL** |
| --- | --- | --- | --- | --- | --- |
| # samples | 65 | 75 | 61 | 80 | 281 |
| Total | 240 | 287 | 233 | 279 | 1039 |
| Males | 207 | 260 | 217 | 246 | 930 |
| Females | 33 | 27 | 16 | 33 | 109 |
| % Males | 86.3 | 90.6 | 93.1 | 88.2 | 89.5 |

Capture numbers and percentage ratio of males to females during three month seasons of the year over five years are compared. Sample numbers were similar for each season and the % male value range from 86.3 – 93.1% did not vary greatly from the mean value of 89.5%. There is no evidence for seasonality in male or female capture bias.
