# Supplementary material for: Nautilus pompilius Life History and Demographics at the Osprey Reef Seamount, Coral Sea, Australia
Source: PLoS One. 2011 Feb 10;6(2):e16312. doi: 10.1371/journal.pone.0016312 (PMC3037366; doi:10.1371/journal.pone.0016312)
Supplement: Table S2 — Small mesh trapping capture results. Twin traps comprising one large mesh (75×50 mm) and one small mesh (30×30 mm) trap ‘piggy backed’ together were deployed on 41 occasions between 100 to 460 m and failed on all occasions to capture any juvenile Nautilus. The large mesh trap caught immature to mature Nautilus at a rate consistent with overall capture rates. (DOCX) [file pone.0016312.s004.docx]

**Table S2. Small mesh trapping capture results.**

| **Depth (m)** | **# samples** | **# caught** | **# juveniles** |
| --- | --- | --- | --- |
| <150 | 11 | 0 | 0 |
| 150-200 | 0 | 0 | 0 |
| 200-250 | 9 | 84 | 0 |
| 250-300 | 4 | 46 | 0 |
| >300 | 17 | 141 | 0 |
| TOTAL | 41 | 271 | 0 |

Twin traps comprising one large mesh (75x50 mm) and one small mesh (30x30 mm) trap ‘piggy backed’ together were deployed on 41 occasions between 100 to 460 m and failed on all occasions to capture any juvenile *Nautilus*. The large mesh trap caught immature to mature *Nautilus* at a rate consistent with overall capture rates.
