# Supplementary material for: Nautilus pompilius Life History and Demographics at the Osprey Reef Seamount, Coral Sea, Australia
Source: PLoS One. 2011 Feb 10;6(2):e16312. doi: 10.1371/journal.pone.0016312 (PMC3037366; doi:10.1371/journal.pone.0016312)
Supplement: Table S3 — Comparison table of Nautilus demographic studies. A comprehensive summary of field study data for the Nautilus demographic parameters of shell length, number of septa, male: female ratio and percentage of mature individuals is provided. The data comes from a wide range of studies and locations and covers all extant species of Nautilus known to date. (DOCX) [file pone.0016312.s005.docx]

**Table S3. Comparison table of *Nautilus* demographic studies.**

| **Location** | **Species** | **Study** | **# ind.** | **Mean mature diameter**  **(mm)** | | | **#**  **septa** | **% males** | **% mature** | |
| --- | --- | --- | --- | --- | --- | --- | --- | --- | --- | --- |
|  |  |  |  | M + F | Male | Female |  |  |  |  |
| Osprey Reef, Coral Sea, Aust. | N.pompilius | This paper | 2460 | 130.7 | 131.9 | 118.9 | 27 | 89.5 | 58 |  |
| PNG | N.pompilius | Saunders, 1987 | 130 | - | 178.2 | 157.2 | - | 75.2 | 97.7 |  |
| PNG | A.scrobiculatus | Saunders, 1987 | 30 | - | 181.3 | 171.6 | - | 84.0 | 83.3 |  |
| Palau | N.belauensis | Saunders, 1978 | 375 | 204 | 209 | 198 | 35-37 | 72.0 | 58.9 |  |
| Palau | N.belauensis | Kakinuma, 1995 | 94 | - | 204.3 | 193.2 | - | 61.3 | - |  |
| New Britain, PNG | N.pompilius | Willey, 1902 | 216 | - | - | - |  | 70.0 | - |  |
| New Ireland, PNG | N.pompilius | Saunders, 1987 | 22 | 173.3 | 173.5 | 169 | - | 95 | - |  |
| Port Moresby, PNG | N.pompilius | Saunders and Ward | 48 | 151.8 | 152.7 | 137.5 | 27-30 | 94 | - |  |
| N.Sulu Sea, Indonesia | N.pompilius | Saunders, 1984 | 25 | 115.6 | - | - | 26-28 | - | - |  |
| Philippines | N.pompilius | Haven, 1972, 1977 | 3000 | - | - | - | - | 92 | - |  |
| Philippines | N.pompilius | Griffin, 1902 | 66 | - | - | - | - | 77 | - |  |
| Philippines | N.pompilius | Hayasaka, 1983 | 32 | 164 | 171.4 | 161.9 | - | 32.7 | 96.9 |  |
| Fiji | N.pompilius | Ward, 1977 | 46 | 141.6 | 143.4 | 135.5 | 28-29 | 86 | - |  |
| Fiji | N.pompilius | Zann, 1984 | 90 | 133 | - | - | - | 90 | 50 |  |
| Fiji | N.pompilius | Tanabe, 1985 | 31 | 141.3 | 145.2 | 135 | - | 75 | 16 |  |
| Fiji | N.pompilius | Saunders, 1989 | 40 | - | 146 | 136.5 | - | 80 | 55 |  |
| American Samoa | N.pompilius | Saunders, 1989 | 39 | - | 174.5 | 164.9 | - | 79.5 | 28.2 |  |
| New Caledonia | N.macromphalus | Landman, 1988 | 1 | 165 | - | - | 31 | - | - |  |
| New Caledonia | N.macromphalus | Ward, 1980 | 114 | - | - | - | 30-32 | 69 | - |  |
| GBR, Australia | N.pompilius | Saunders, 1987 | 4 | 159.8 | - | - | - | - | - |  |
| GBR, Australia | N.stenomphalus | Saunders, 1987 | 7 | - | 165 | - | - | - | - |  |

A comprehensive summary of field study data for the *Nautilus* demographic parameters of shell length, number of septa, male: female ratio and percentage of mature individuals. The data comes from a wide range of studies and locations and covers all extant species of *Nautilus* known to date.
