# Supplementary material for: Nautilus pompilius Life History and Demographics at the Osprey Reef Seamount, Coral Sea, Australia
Source: PLoS One. 2011 Feb 10;6(2):e16312. doi: 10.1371/journal.pone.0016312 (PMC3037366; doi:10.1371/journal.pone.0016312)
Supplement: Table S4 — Comparison table of Nautilus growth rate studies. Growth, embryonic development period and age estimation data are summarized from field (F), aquarium (A) and radiometric / geochemical (R) studies in the literature. Note the similarity of age estimates for both this study and that of Saunders, the only field-based studies of growth rate. (DOCX) [file pone.0016312.s006.docx]

**Table S4. Comparison table of *Nautilus* growth rate studies.**

| **Study** | **Species** | **Nautilus origin** | **Study** | **# spec.** | **Stage** | **Rate of apertural growth (mm/day)** | | | **Embryonic period**  **(days)** | **Age to maturity estimation (yrs)** |
| --- | --- | --- | --- | --- | --- | --- | --- | --- | --- | --- |
|  |  |  |  |  |  | Mean | Range | |  |  |
| This study | N.pomp | Aust. | F | 15 | I - S | 0.061 | | 0.018 – 0.111 | - | 14.5 - 16.5 |
| Ward (1983) | N.pomp | Phil. | A | 4 | I | 0.23 | | 0.15 – 0.25 | - | - |
| Landman(1989) | N.pomp | PNG | R | 2 | M | - | | - | - | - |
| Landman(1989) | A.scrob | PNG | R | 4 | M | - | | - | - | - |
| Landman(1989) | N.pomp | Phil. | A | 4 | I | 0.24 | | 0.19 – 0.31 | - | - |
| Landman (1989) | N.pomp | PNG | R | 1 | I | 0.12 | | 0.12 | - | - |
| Ward (1985) | N.pomp | Phil. | A | 10 | I | 0.40 | | 0.06 – 0.58 | - | - |
| Ward (1985) | N.macro | N. Cal | A | 2 | I | 0.40 | | 0.03 – 0.72 | - | - |
| Ward 1981 | N.macro | N. Cal | A | 44 | I | 0.14 | | 0.09 – 0.175 | - | - |
| Cochran, 1984 | N.pomp | Palau | R | 7 | I - M | 0.12 | | 0.10 - 0.14 | - | 10+ |
| Kanie, 1979 | N.macro | N. Cal | A | 7 | I - M | 0.14 | | 0.14 | - | - |
| Landman (1987) | N.pomp | Phil. | R | 2 | I | 0.21 | | 0.20 – 0.22 | - | - |
| Landman,(1988) | N.macro | N. Cal | R | 1 | M | - | | - | - | 10-12 |
| Westermann, 2004 | N.pomp | Phil. | A | 9 | J, I, S | 0.124 | | 0.124 | - | 7.3-8 |
| Martin (1978) | N.macro | N. Cal | A | 7 | I | 0.25 | | 0.17 – 0.30 | - | - |
| Saunders 1984 | N.pomp | Palau | F | 7 | I - M | 0.12 | | 0.04 – 0.12 | - | 14.5-17.2 |
| Hamada, 1987 | N.macro | N. Cal | A | 6 |  | 0.08-0.127 | | 0.08-0.127 | - | - |
| Uchiyama, 1996 | N.macro | N. Cal | A | 19 | H | - | | - | 269 - 362 | - |
| Okubo, 1995 | N.pomp | Palau | A | 3 | H | 0.24 | | 0.20 – 0.28 | - | - |
| Arnold, 1990 | N.pomp | Palau | A |  | H | - | | - | 305 | - |

Growth, embryonic development period and age estimation data are summarized from field (F), aquarium (A) and radiometric / geochemical (R) studies in the literature. Note the similarity of age estimates for both this study and that of Saunders, the only field-based studies of growth rate.
